# A Spatio-Temporal Analysis of OECD Member Countries’ Health Care Systems: Effects of Data Missingness and Geographically and Temporally Weighted Regression on Inference

**DOI:** 10.3390/ijerph20136265

**Published:** 2023-06-30

**Authors:** Peter Akioyamen, Mehmet A. Begen

**Affiliations:** 1 Statistics & Actuarial Sciences, Western University, London, ON N6A 5B7, Canada; pakioyam@uwo.ca; 2 Computer Science, Western University, London, ON N6A 5B7, Canada; 3 Epidemiology & Biostatistics, Western University, London, ON N6A 5B7, Canada; 4 Ivey Business School, Western University, London, ON N6G 0N1, Canada

**Keywords:** OECD, health system performance, global health, imputation, geographically and temporally weighted regression, spatio-temporal analysis

## Abstract

Determinants of health care quality and efficiency are of importance to researchers, policy-makers, and public health officials as they allow for improved human capital and resource allocation as well as long-term fiscal planning. Statistical analyses used to understand determinants have neglected to explicitly discuss how missing data are handled, and consequently, previous research has been limited in inferential capability. We study OECD health care data and highlight the importance of transparency in the assumptions grounding the treatment of data missingness. Attention is drawn to the variation in ordinary least squares coefficient estimates and performance resulting from different imputation methods, and how this variation can undermine statistical inference. We also suggest that parametric regression models used previously are limited and potentially ill-suited for analysis of OECD data due to the inability to deal with both spatial and temporal autocorrelation. We propose the use of an alternative method in geographically and temporally weighted regression. A spatio-temporal analysis of health care system efficiency and quality of care across OECD member countries is performed using four proxy variables. Through a forward selection procedure, medical imaging equipment in a country is identified as a key determinant of quality of care and health outcomes, while government and compulsory health insurance expenditure per capita is identified as a key determinant of health care system efficiency.

## 1. Introduction

Assessing determinants of health care quality and efficiency is of importance to policy-makers and public health officials. The identification of factors contributing to the improvement or degradation of health care system performance can inform regulatory and funding decisions over time.

In the past, limited attention has been given to imputation schemes and their use in this context. Most studies neglect to mention which methods they employ and why. Yet, in practice, data are rarely complete and the imputation method used results in a fundamentally different data set on which statistical inference is conducted. We use data from the Organisation for Economic Co-operation and Development (OECD) to investigate the relationship between key health indicators and the efficiency of health care systems along with the health outcomes and quality of care provided globally. Specifically, we use the average length of stay in inpatient care as a proxy for health care system efficiency, while life expectancy, infant mortality, and potential years of life lost, a measure of premature mortality, are used as proxy variables for health outcomes and quality of care. Here, we show the effect that imputation can have in statistical modeling, introducing variation in regression coefficient estimates and greater uncertainty in statistical inference. Additionally, we suggest that many of the models used in previous works are likely ill-specified. OECD data are recorded across geographical regions and time periods. Many linear models used have not accounted for spatial or temporal variation in the data, leading to bias in coefficient estimates and standard errors. Though encoding geographical information in a linear model through dummy variables may produce a high performing model, it fails to account for the effects of geographic proximity and variation that may influence the set of covariates studied. The use of interaction terms may assist in this respect, but is likely to decrease the interpretability of the model, making the primary goal of statistical inference difficult. To the best of our knowledge, researchers have yet to analyze the spatio-temporal variation in determinants and their impact on health care system performance. While temporal autocorrelation refers to the relationship between successive observations of a variable, spatial autocorrelation refers to the presence of systematic spatial variation in a mapped variable. Where adjacent observations have similar data values, positive spatial autocorrelation exists, and contrasting values indicate negative spatial autocorrelation. The presence of autocorrelation implies information redundancy, and spatial autocorrelation in particular has important implications for the methodology of spatial data analysis. Geographically and temporally weighted regression (GTWR) is used in this study to simultaneously address the spatial and temporal autocorrelation in the data, allowing for more robust modeling and inference.

### 1.1. Existing Work

In the past, comparisons of health care systems have been made from a clinical perspective. With the growth of analytics, more statistical modeling and computational methods have been used to conduct rigorous analysis, provide robust recommendations, and draw insights out of data. Refs. [1,2] analyze OECD data to understand why United States health care spending has exceeded that of other developing nations in the past. Ref. [3] compare health care systems internationally, discussing spending, supply, and utilization for specific categories of health care services—the authors also present some benefits and drawbacks in using OECD data. Studies have explored the use of Data Envelopment Analysis techniques to quantify and comparatively evaluate the efficiency of health care systems. Employing the dynamic network and window analysis approaches, respectively, refs. [4,5] quantify efficiency and develop ordinal rankings of OECD health care systems. Differing in prescribed methodology, collectively the studies are inconclusive, diverging in their determination of the most and least efficient health care systems. Ref. [4] overlays qualitative findings related to public and private health infrastructure as well as health policy to further explain findings in health care system efficiency and the levels of individual variables used in the analysis. Clustering has also been used to study systems as researchers attempted to understand health care system performance and categorize 27 OECD countries into four distinct profiles [6]. Ref. [7] uses machine learning to predict healthcare expenditures of OECD countries. Ref. [8] investigate factors influencing key health outcomes—using life expectancy and infant mortality as proxies—across 25 OECD countries from 1990–2002. The authors note that previous related works are limited in scope (either time horizon or number of countries considered) or analysis approach (primarily qualitative). The authors included some social factors in their analysis and found that hospital bed days, followed by the supply of physicians and education level were influential factors for life expectancy, while physician supply, immunization and education were statistically significant factors for the regression of infant mortality. The results from [8] agree with the common perception that lowering mortality rates requires an emphasis on pre- and post-natal care. Ref. [9] analyze OECD health care data to identify determinants of life expectancy across 19 countries over a 15-year time span, finding that doubling annual pharmaceutical expenditures adds about one year of life expectancy for males at age 40 and slightly less than a year of life expectancy for females at age 65.

Missing data are ubiquitous in statistical analysis and health-related data. There are many data imputation methods, each with a common goal of estimating values for missing, invalid, or inconsistent data. Discussion regarding the treatment of imputation and data missing mechanisms in the aforementioned works is minimal. It is known that sub-optimal strategies for missing values can produce biased estimates and invalid conclusions [10,11]. The severity of these consequences may increase based on the mechanism by which missing data has been generated, the specific imputation method used, and the proportion of missing data present.

In addition, statistical modeling on OECD data has been primarily restricted to multivariate linear regression or related variants. The inability for this class of methods to account for the spatial relationships between observations in the data presents a unique challenge that has not been addressed in this context. Geographically weighted regression (GWR), an extension of ordinary least squares, allows the relationships between the independent and dependent variables to vary by spatial locality—effectively taking spatial non-stationarity into consideration [12,13]. Ref. [14] use GWR to investigate local variations in the association between immunization coverage and selected socioeconomic and demographic determinants across census area units (CAU) within New Zealand. The authors found that childhood immunization coverage varies by socioeconomic and demographic factors across CAUs with nearly all explanatory variables exhibiting spatial variation in their association with immunization coverage. GWR has also been used to study infant mortality rates across prefectures in China [15]. The authors use GWR to examine the spatial variations of the relationships between economic and health care factors and infant mortality rate, finding spatial heterogeneity through three key variables. Geographically weighted regression has been applied in the epidemiological modeling of disease prevalence, demographic factors, and health outcome disparity at the country-wide level, but has not been used to conduct statistical inference on the performance of country-wide health care systems globally.

Geographically and temporally weighted regression accounts for both spatial and temporal non-stationarity by incorporating temporal effects into GWR; dealing with spatial and temporal variation, GTWR has been shown to outperform GWR and temporally weighted regression (TWR) [16]. In [17], GTWR is used to study spatial heterogeneity and uncover the contributions of various social determinants of health in geographic variation of premature mortality in Taiwan. The study found that two variables, ethnicity and education, were able to explain significant amounts of geographical variation in premature mortality, and further, suggested that tailored health programs are needed to improve social determinants of health in areas most likely to exhibit premature mortality. Other applications of GTWR exist in the study of transit ridership and air pollution [18,19,20], but the application of geographically and temporally weighted regression in the context of public health and health care data is extremely limited. It is possible that the exclusion of spatial and temporal factors in previous analyses may have resulted in misleading or otherwise incomplete analyses.

### 1.2. Motivation and Contributions

The lack of explicit discussion about data missingness and imputation coupled with potential model misspecification in analysis can contribute to statistical inference that is misinformed and misleading. Imputation methods applied in this work are discussed and used to illustrate how a lack of transparency can be detrimental to reproducibility and inference through unstable regression coefficients and variation in model predictive capability.

GTWR is established as a viable method of statistical analysis for spatio-temporal health data, with notable inferential benefits. Specifically, we identify the number of medical imaging units in a country as a strong determinant of health outcomes and quality of care and we find that in terms of $AIC_{c}$, adjusted $R^{2}$, and likelihood, GTWR outperforms linear regression, indicating that GTWR is preferable in the analysis of OECD and related data. Similar to a subset of past works, in this study we center the analysis on OECD data that span multiple countries and points in time. We take a unique approach to the analysis of these data by actively adjusting for spatial and temporal autocorrelation through GTWR, which to the best our knowledge has not been carried out previously. Common health statistics are used collectively as proxies for health care system performance as we seek to identify factors influencing these systems internationally.

## 2. Methods

### 2.1. OECD Data

The data we use from the OECD database consist of frequently requested key health statistics recorded from 1960–2019. A subset of the data in the OECD database was compiled and used in this work, comprised of 26 variables and 399 observations, with a total of 501 missing values. Four health statistics are treated as dependent variables in individual models. Each dependent variable acts as a proxy for a specific property in a national health care system. A spatio-temporal component is embedded within the data through the Year and Country variables, indicating the year of the recorded observation and its country of origin. The remaining 20 variables used in this analysis were selected based on the understood relations they may have with the target variables. A total of 21 countries were included in the analysis: Australia, Austria, Belgium, Canada, Czech Republic, Denmark, Finland, France, Germany, Hungary, Iceland, Ireland, Japan, South Korea, Latvia, Lithuania, Netherlands, Poland, Slovakia, Slovenia, and Spain. The selection of countries considered was determined by criteria that include sufficient economic development such that comparison and inference between countries is meaningful, and a maximum missingness threshold of 15% for any given statistic per country, to ensure that any imputation methods used in this work have a basis to make reasonable estimates of missing values. Though the original data ranges from 1960 to 2019, we truncate the analyzed time period to one that is more representative of modern economic conditions (2000–2018) to strengthen the extent to which statistical inference generalizes while avoiding severe covariate shift. A list of all variables used in this work along with descriptions can be found in Table A1 and Table A2 within Appendix A.

### 2.2. Imputation

We employ four imputation schemes to address missingness in the data set. In particular, complete case (CC) analysis, K-Nearest Neighbors (KNN) imputation [21,22], mean imputation, and multiple imputation by chained equations (MICE) [23,24] are used. Single imputation methods such as KNN and mean imputation are those that use one value to fill a missing data element, without defining an explicit model for the partially missing data [25]. In these methods the imputed values are estimates which have standard errors. Single imputation does not reflect the uncertainty in the predicted missing values and ignores the fact that no imputation method can provide the true value precisely. Even if the data are missing completely at random (MCAR), the method can lead to severely biased estimates in regression [26]. Multiple imputation is widely preferred over other methods of dealing with data missingness due to its empirical and theoretical properties [27]. MICE attempts to account for the uncertainty in the imputed values, using several complete data sets to provide both within-imputation and between-imputation variability [28], a characteristic which makes multiple imputation preferable over single imputation in most circumstances.

Complete case analysis only includes observations for which there are no missing data elements. The disadvantages of this approach stem from the potential information loss and bias when missing data are not MCAR, though it can be applied without modifications to data so its simplicity can be beneficial [29]. It has been shown by [30] that when data are MCAR both complete case analysis and multiple imputation have negligible bias. When data are missing at random (MAR), showing systematic differences between the missing and observed values that can be explained by other observed variables, CC is biased towards the null and underestimates standard errors of coefficients. However, when missingness is independent of the outcome given the covariates, CC has negligible bias and multiple imputation is biased away from the null, overestimating the standard errors. With more general missing data mechanisms, bias tends to be smaller for multiple imputation than for complete case analysis. Due to the potential presence of bias in the standard errors of coefficient estimates in regression, it may be misleading to compare the standard errors resulting from these two methods. Complete case analysis yielded four data sets, which have 198 observations for average length of stay, 199 observations for infant mortality and life expectancy, respectively, and 182 observations for potential years of life lost. No observations of Japan remained in the complete case data sets. As a result, all linear regression models fit to complete case data only consider 20 countries and encode geographical information as dummy variables. The other imputation methods all result in 399 observations. The data are split into training and testing sets stratified by country, with 60% of observations in the training set. Imputation results from MICE are averaged over 20 imputed data sets and model coefficients are pooled using Rubin’s rules [27].

### 2.3. Geographically and Temporally Weighted Regression

Geographically weighted regression (GWR) models are defined as,

(1) $$\begin{array}{cc} \; & Y_{i}=\beta_{0}\left(u_{i},v_{i}\right)+\sum_{k}\beta_{k}\left(u_{i},v_{i}\right)X_{ik}+\epsilon_{i}\;\;\;i=1,\ldots ,n \end{array}$$

where $\left(u_{i},v_{i}\right)$ denotes the coordinates of the point *i* in space, $\beta_{0}\left(u_{i},v_{i}\right)$ represents the intercept value, and $\beta_{k}\left(u_{i},v_{i}\right)$ is a set of values of parameters at point *i*. Unlike the ‘fixed’ coefficient estimates over space in what is considered a ‘global’ linear regression model, GWR allows parameter estimates to vary across space and is therefore likely to capture local effects. As an extension of GWR, geographically and temporally weighted regression is used to address both spatial and temporal nonstationarity in the data. GTWR can be expressed as,

(2) $$\begin{array}{cc} \; & Y_{i}=\beta_{0}\left(u_{i},v_{i},t_{i}\right)+\sum_{k}\beta_{k}\left(u_{i},v_{i},t_{i}\right)X_{ik}+\epsilon_{i}\;\;\;i=1,\ldots ,n \end{array}$$

where the goal is to produce estimates of $\beta_{k}\left(u_{i},v_{i},t_{i}\right)$ for each variable *k* and each space–time location, with a point *i* representing an observation in the data set.

A kernel provides weightings used to calibrate the model at each location, generally decreasing the weight of peripheral observations. It has been shown that the shape of the kernel has minimal impact on the results in geographically weighted regression [31]; the difference in results produced by the bisquare kernel and the Gaussian kernel was negligible in this analysis as well. We employ a Gaussian kernel defined as

$$\begin{array}{c} w\left(d_{ij}\right)=e^{-\frac{d_{ij}^{2}}{2h^{2}}} \end{array}$$

where $d_{ij}$ is the distance between the point *i* being estimated and the point *j* being weighted. The capital city of each country is used to define the points used in the computation of distance. Non-negative parameter *h* is the bandwidth, which defines the size of the weighting window. For kernels that assign zero weights, it determines the distance beyond which this assignment occurs. As h⟶∞, the coefficient estimates converge towards the global OLS model, which equates to fitting a regular linear regression to the data. The Gaussian kernel provides weights to all observations, tending to zero as the distance between points *i* and *j* exceeds the selected bandwidth.

A forward selection procedure is used to construct each model, reducing the number of variables by considering the minimization of the corrected Akaike Information Criterion ($AIC_{c}$) at each iteration. $AIC_{c}$ is computed as specified by [13] using the effective number of parameters in a given GTWR model and is preferred due to the small sample size across all models relative to the maximal number of effective parameters [32]. For each set of predictors, the optimal value for *h*, the bandwidth parameter, is computed according to [16,33], and is then used to fit the model.

## 3. Results

### 3.1. Linear Regression Analysis

We develop an independent linear regression formulation for each proxy variable, resulting in a model for average length of stay in inpatient care, infant mortality, potential years of life lost, and life expectancy at birth, respectively:

(3) $$\begin{array}{cc} \; & Y_{\mathrm{AverageStay}}=X_{i}\beta_{AS}+\epsilon \end{array}$$

(4) $$\begin{array}{cc} \; & Y_{\mathrm{InfantMortality}}=X_{i}\beta_{IM}+\epsilon \end{array}$$

(5) $$\begin{array}{cc} \; & Y_{\mathrm{PYLL}}=X_{i}\beta_{PYLL}+\epsilon \end{array}$$

(6) $$\begin{array}{cc} \; & Y_{\mathrm{LifeExpectancy}}=X_{i}\beta_{LE}+\epsilon \end{array}$$

where ϵ is a normally distributed random error, $X_{i}$ is the imputed data matrix with i=1,2,3,4 representing a given method of imputation, and β is the vector of coefficients to be estimated using least squares. Four models are fit for each dependent variable, one for every imputation method considered. Table 1 displays the resulting regression coefficient estimates by an imputation method with respect to each of the response variables considered. The standard deviation of the estimates is also displayed. For each response variable, there is a subset of predictors that exhibit large standard deviation relative to the scale of the response variable. In each case, the estimated intercept value shows notable variation, impacting the ability to confidently infer the effect on the response given that an observation corresponds to the base country, Australia. In the absence of explicit statements regarding the data missingness mechanism and the imputation methodology, it becomes increasingly difficult to make statistical inference generalizeable, and reproducibility becomes a challenge. Recommendations concerning health policy based on related findings or comparison between coefficient estimates, such as the effect of a country on a selected proxy variable seen here, are clearly weakened. The potential bias embedded in coefficient estimates due to an inappropriate selection of imputation method or a vast misspecification in the data missingness mechanism assumed in the analysis may only exacerbate the misinterpretation that results from the statistical analysis.

**Figure 1:**
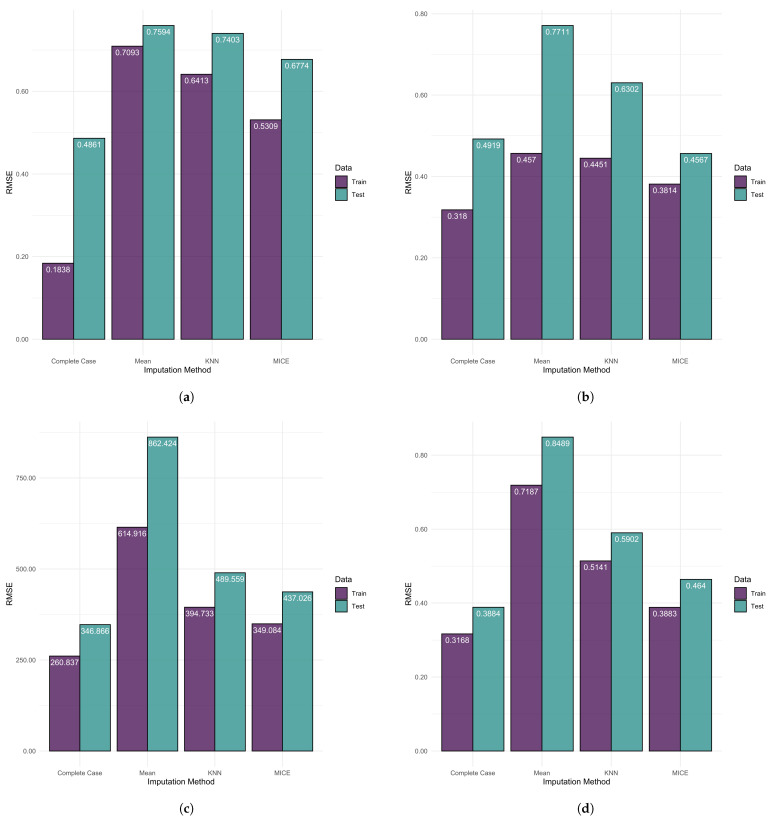
Linear regression model performance for each imputation method grouped by response variable. (**a**) Average length of stay in inpatient care. (**b**) Infant mortality rate. (**c**) Potential years of life lost. (**d**) Life expectancy of the total population at birth.

The impact of imputation on model performance as assessed through root mean square error (RMSE) is shown for all response variables in Figure 1. The complete case model outperforms the other imputation methods across all proxy variables, while MICE imputation consistently achieves the second lowest RMSE. Of the four methods considered, models trained using mean imputation have the highest RMSE. There is notable variation in model performance across the imputation methods. The variation in model performance along with the instability in coefficient estimates heightens the uncertainty behind any inference and determination of predictor impact derived from the use of linear models when the imputation methodology is not discussed.

**Figure 2:**
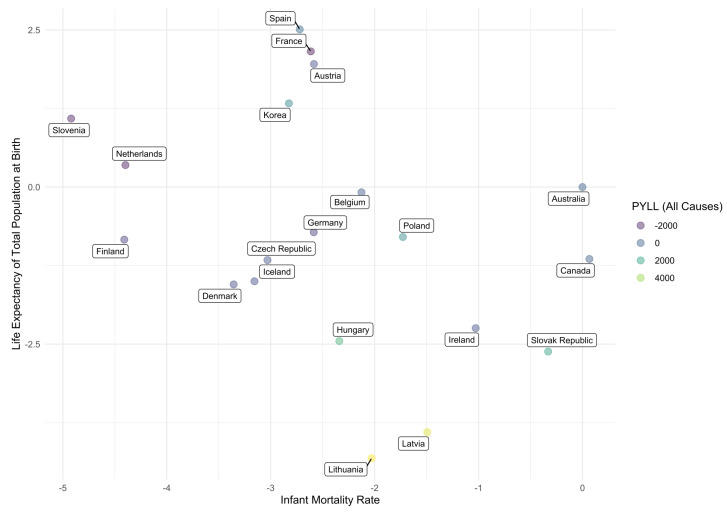
Relative effect of location on quality of care and outcomes in health care systems.

For potential years of life lost, infant mortality, and life expectancy, we extract the dummy variable coefficient estimates for OECD member countries from models trained on complete case data. With Australia as the baseline country, these coefficient estimates indicate the relative change in the respective proxy variable given that an observation is associated with a specific country. We may expect countries in close geographical proximity to have similar coefficient estimates for a particular response variable. The expectation is evaluated empirically by showing how these coefficient estimates vary together. In Figure 2, countries are labeled and naturally occurring clusters of countries can be identified. Despite the inability for the linear regression models to account for spatial autocorrelation directly, within some clusters it is evident that the geographical distance between countries is minimal, coinciding with what we expect to observe. The Baltic countries Lithuania and Latvia, seen in the bottom-central area of Figure 2, have a similar effect on the quality of care and outcomes through the three proxy variables. In the top-central area, Spain and France, which are also adjacent to one another, appear fairly close. These countries seem to share a similar relative effect on life expectancy and infant mortality, but diverge in their effect on potential years of life lost. Czech Republic, Poland, and Germany border each other, while Belgium borders Germany; all four countries appear in the same area of the figure, indicating similar effects on life expectancy and infant mortality. Poland differs most significantly from the other countries in its effect on potential years of life lost.

Using statistical tests for spatial and temporal autocorrelation, we formalize this finding (Table 2). Moran’s I is employed to test for spatial autocorrelation in each proxy variable while the Durbin–Watson and Breusch–Godfrey tests are used to assess the presence of temporal autocorrelation of order 1 [34,35,36]. All tests were conducted at α = 0.05 and indicated the true autocorrelation in the data, spatial and temporal, is nonzero.

### 3.2. Spatio-Temporal Analysis

An independent GTWR model is constructed for each proxy variable using complete case analysis to address data missingness. As a result, Japan is not considered in any of the following models and each model is fit to all observations in the respective data set. A summary of the GTWR coefficient estimates is shown in Table 3.

**Figure 3:**
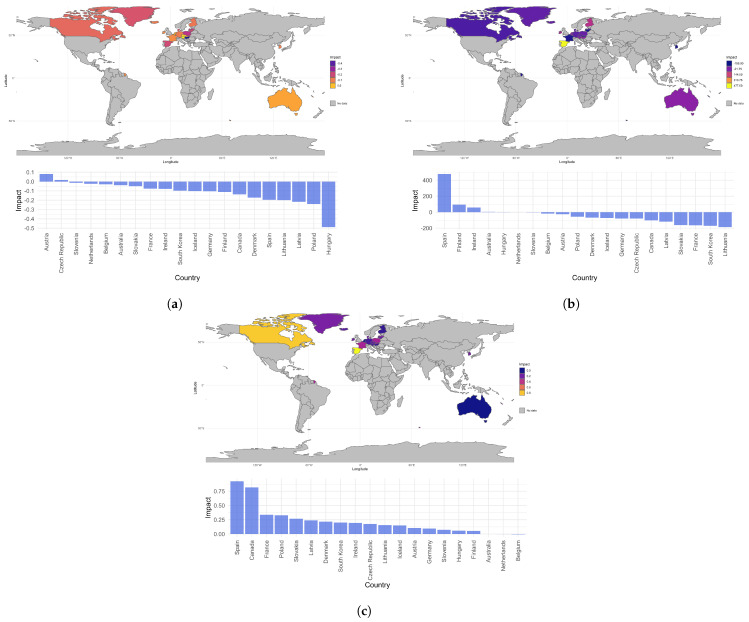
Geographical distribution of imaging equipment’s impact on quality of care as estimated by GTWR models. (**a**) Median GTWR coefficients for count of CT scanners in $Y_{\mathrm{InfantMortality}}$. (**b**) Median GTWR coefficients for count of CT scanners in $Y_{\mathrm{PYLL}}$. (**c**) Median GTWR coefficients for MRI units in $Y_{\mathrm{LifeExpectancy}}$.

Increasing government and compulsory health insurance schemes expenditure per capita generally decreases the average length of stay in inpatient care across health care systems globally, having a positive effect on health care system efficiency. Based on the median estimated impact, increases in alcohol consumption tend to increase the average length of stay, though this relationship does not exhibit the same level of homogeneity across countries. Producing more medical graduates seems to decrease the potential years of life lost in the population while the number psychiatric care beds per 1000 people exhibits both positive and negative effects on quality of care and outcomes produced by health care systems depending on the geographical location. Increasing the number of medical imaging units (CT scanners or MRIs) seems to increase the quality of care and outcomes in a country. As the total number of CT scanners per million people increases, potential years of life lost and infant mortality in a location tend to decrease, as indicated by approximately three quarters of the local coefficient estimates for both GTWR models (Table 3b,c). The inverse relationship is seen with respect to total MRI units per million people and the life expectancy in a country. More than three quarters of the local coefficient estimates are positive, indicating that most locations tend to see an increase in life expectancy as the number of MRI units increases. The median coefficient estimate is computed by country and plotted, producing the geographical distributions seen in Figure 3. Few countries experience a negative effect on infant mortality when increasing the number of CT scanners or a negative effect on life expectancy when increasing the number of MRI units (Figure 3a,c). The magnitude of any negative impact is estimated to be minimal in applicable countries. An increase in the number of CT scanners also seems to have a largely positive impact on the potential years of life lost, decreasing the value in most countries, though, Spain is identified as an outlier since it is estimated to experience a substantial increase in potential years of life lost for every additional MRI unit (Figure 3b). Across the majority of the central European region, we observe a similar impact in increasing the number of medical imaging units on the quality of care and outcomes produced by health care systems, regardless of the proxy variable considered. The effect of increasing the number of CT scanners on infant mortality and potential years of life lost is comparable in Iceland and Canada as well.

**Figure 4:**
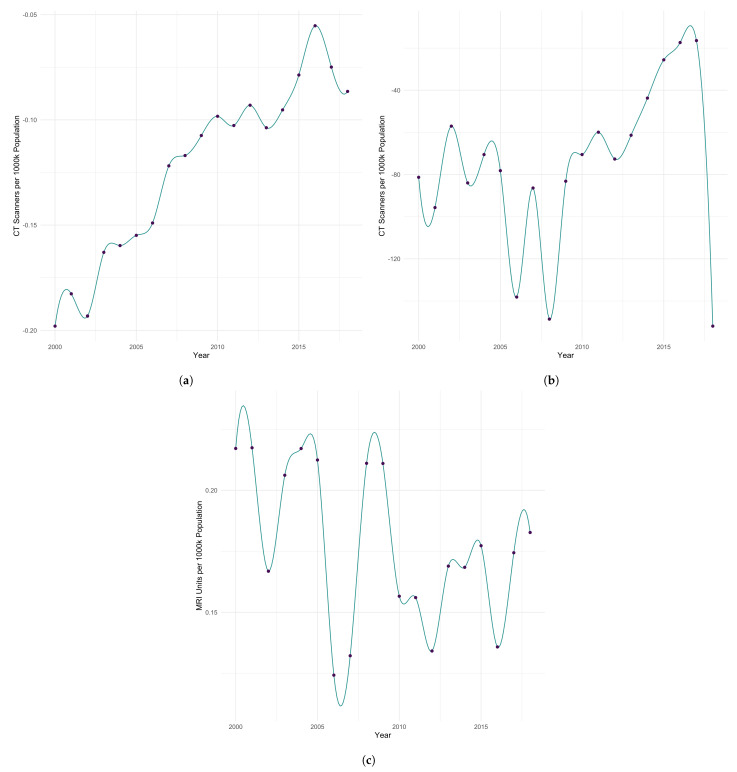
Variation in the median impact of medical imaging units on health care system quality of care and outcomes over time as estimated by GTWR. (**a**) Median coefficient estimates for CT scanners with infant mortality as the response variable. (**b**) Median coefficient estimates for CT scanners with potential years of life lost as the response variable. (**c**) Median coefficient estimates for MRI units with life expectancy of the total population as the response variable.

To assess the temporal variation in the estimated impact of medical imaging units on health care system quality of care and outcomes, we compute the global median coefficient estimates for medical imaging units in the aforementioned three GTWR models at each point in time considered in this work. The splines fit to the resulting data can be seen in Figure 4. In Figure 4a, the median effect of CT scanners on infant mortality is shown. Although additional CT scanners tend to decrease infant mortality, the median coefficient estimate has become less negative over time, indicating that the positive impact of an additional CT scanner on the quality of care and outcomes has weakened since 2000. It is likely that there are other contributing factors to this phenomenon, warranting further investigation. The median coefficient estimates for the impact of CT scanners on potential years of life lost exhibits a similar trend from 2012–2016 in which the median coefficient estimates increase, becoming less negative, indicating that the magnitude of the median decrease in potential years of life lost attributable to an additional CT scanner in a country is smaller than that estimated previously (Figure 4b). Though, this trend breaks after 2016. Within Figure 4b, we otherwise see a positive influence on quality of care and outcomes in health care systems by decreasing potential years of life lost globally through time. The effect of the number of MRI Units on the quality of care and outcomes across health care systems has remained generally positive from 2000–2018, as it is estimated to have had the consistent effect of increasing life expectancy by varying amounts throughout this time (Figure 4c).

Table 4 displays a comparison of the goodness-of-fit of OLS models, both with and without country dummy variables, against GTWR. In all cases, the final GTWR model shows significant improvement relative to the OLS model that does not incorporate the dummy variables with respect to both $AIC_{c}$ and adjusted $R^{2}$. With the exception of infant mortality, the GTWR model achieves a notable improvement in model fit compared to modeling the response using OLS with dummy country variables. The conditional probability of GTWR being the correct model for each response variable is assessed through Akaike weights. Geographically and temporally weighted regression has a higher likelihood of being the correct specification relative to the linear regression configurations that may be used traditionally to model the performance of health care systems with OECD data. Explaining 94.01% of the variation in the response variable compared to 92.73%, GTWR improves upon the next best model marginally in $AIC_{c}$, while adjusted $R^{2}$ and the likelihood of GTWR is less definitive in the modeling of infant mortality.

## 4. Discussion

We investigated the relationship between key health indicators and their impact on the efficiency of health care systems along with health outcomes and quality of care produced globally. More specifically, average length of stay in inpatient care is used as a proxy variable for health care system efficiency, while infant mortality, potential years of life lost, and life expectancy are used as proxy variables for health outcomes and quality of care. Geographically and temporally weighted regression is used to account for spatial and temporal autocorrelation in the data. A forward selection procedure is employed and important variables are selected based on $AIC_{c}$. The number of medical imaging units, either CT scanners or MRIs, was commonly identified as an important variable in modeling health outcomes and quality of care through the proxy variables. The coefficients for the variable coincided with the expected sign; the geographical variation in coefficient estimates for medical imaging units provided insight into the largely positive impact increasing the number of units in a country has on its health outcomes and quality of care. Through regression analysis [8] found medical technology to be a statistically significant predictor of life expectancy, aligning with the importance of the MRIs and CT Scanners shown in this work. We also found that health care expenditure was statistically significant in predicting life expectancy, but not infant mortality. Previous work has shown that health care expenditure does not easily translate into human resources [37], which often influences the quality of care and efficiency, supporting the lack of significance found in variables characterizing expenditure in our study and past work with respect to infant mortality. It is important to note that [8] did not consider any spatial or temporal variation in their conducted analysis. There are limited and few previous papers when considering the impact of social determinants and expenditure in relation to quality of care and health care system efficiency.

Japan’s exclusion from GTWR due to data limitations prompts important considerations. While it is generally known that Japan has a significant number of MRIs per person relative to other countries and a high length of stay, the country also has one of the lowest infant mortality rates, highest life expectancy levels, and is often regarded as having one of highest quality health care systems in globally. Cultural preferences impact clinical and surrogate decision making across geographic regions [38,39], yet this is lost in health care data and influences proxy variables such as average length of stay. This implies that qualitative insights from country-specific contexts may enhance the understanding of the relationships between the dependent and independent variables considered. In addition, it may be beneficial to develop more analytically rigorous definitions and measures for health care system efficiency, avoiding circumstances in which conclusions about public health and health care systems derived from quantitative methods contradict well established general knowledge. Employing a hybrid methodology in which the dependent variable acting as a proxy for health care system efficiency or quality of care is derived from Data Envelopment Analysis, for example, and combined with GTWR, may yield insightful results.

GTWR provided a significant improvement over linear regression models that did not encode countries as dummy variables, explaining more variation across all proxy variables. A similar result is observed over linear regression models which did use dummy variables; however, the improvement in explained variation is marginal in the modeling of infant mortality. We show that geographically and temporally weighted regression is a preferable alternative to linear regression for statistical modeling and inference of OECD health care data, which spans both space and time.

Missing data mechanisms are discussed and various imputation methods are used to address data missingness. By varying the imputation method used to treat OECD data, we find that substantial variation results in both model predictive capability and regression coefficient estimates. Empirically, we have shown that in the absence of explicit statements regarding imputation and data missingness, it becomes difficult to validate and understand predictive performance, inference, and recommendations that may result from an analysis of OECD data.

A known limitation of this work is the inability to use MICE for imputation in the geographically and temporally weighted regression analysis. The constraint exists that governs the pooling of coefficient estimates and standard errors when MICE is used in linear regression, not being extended to the spatio-temporal domain. To the best of our knowledge, there is no equivalent of Rubin’s rules that would allow for the aggregation of coefficient estimates across various GTWR models. Due to the many issues with single imputation methods, we elect to use complete case analysis in place of MICE. However, this presents its own set of limitations as information is lost when discarding observations and bias introduced into GTWR coefficient estimates is dependent on the data missingness mechanism.

### Future Works

With a limited number of observations used in the modeling of each proxy variable, little can be stated regarding health policy recommendations. The time horizon that was considered here and the set of countries analyzed should be expanded; future analysis may also consider more health indicators aggregated by the OECD in addition to those studied here as predictors. An expansion in the scope of study in this way will likely introduce more missing data, further emphasizing the need for appropriate model specification in analysis and transparent communication with respect to the treatment of missing data in literature.

Currently, the number of studies which apply geographically and temporally weighted regression for modeling and inference is limited. Despite being an extension of geographically weighted regression, it is yet to be determined if the statistical tests and inferential methods developed for GWR can be extended directly to GTWR in the spatial–temporal domain; this is an area of ongoing study. In geographically weighted regression, it is known that multicollinearity between the coefficients may be responsible for instability in coefficient estimates resulting in a change of sign when adding a new variable into the regression, the counter-intuitive sign of a regression coefficient, or high standard errors of model parameters [40]. Techniques to assess the presence of multicollinearity and reduce its impact in GTWR are limited both in theoretical study and software implementation. Research in this area may simultaneously concern itself with the discovery of more robust variable selection procedures in the spatio-temporal domain.

## 5. Conclusions

We found the presence and use of medical technology to be a key factor of influence in the quality of care and an indicator of health care system efficiency. We think that the results of our study will motivate greater transparency with respect to data imputation methods, enable further exploration of spatio-temporal analysis in public health, and contribute to the development of new hypotheses for studies focusing on the performance of global health care systems.

## Figures and Tables

**Table 1:** Comparison of model coefficients estimates and standard errors by imputation method.

Variable	Average Stay					Infant Mortality					PYLL					Life Expectancy				
	**CC**	**M**	**KNN**	**MICE**	** σ **	**CC**	**M**	**KNN**	**MICE**	** σ **	**CC**	**M**	**KNN**	**MICE**	** σ **	**CC**	**M**	**KNN**	**MICE**	** σ **
Intercept	8.52	−10.5	−4.57	1.44	8.13	5.98	1.59	3.55	12.1	4.57	6151	419	−5088	290	4589	74.9	87.3	84.9	77.9	5.82
CurrExpendGDP	−0.008	−0.325	−0.225	0.114	0.200	0.268	−0.000	0.050	0.108	0.012	64.4	−217	9.00	124	149	−0.120	−0.131	−0.118	−0.129	0.006
CurrExpendUS	−0.001	0.004	0.004	0.003	0.005	0.000	0.003	0.003	0.001	0.001	1.25	4.12	3.82	2.29	1.35	0.002	−0.001	−0.001	0.000	0.001
GovtCompInsCurr	−0.025	0.193	0.096	0.089	0.095	−0.036	0.080	0.035	−0.099	0.078	−14.8	71.2	88.4	−14.6	55.0	0.067	−0.091	−0.040	0.058	0.077
GovtCompInsUS	0.000	−0.006	−0.004	−0.004	0.003	0.001	−0.002	−0.002	0.000	0.001	−0.37	−2.38	−3.11	−1.30	1.21	−0.002	0.002	0.001	−0.000	0.002
OOPPaymentsCurr	−0.111	0.225	0.136	0.092	0.142	0.079	0.195	0.163	−0.010	0.092	−47.5	213	175	52.9	119	0.075	−0.041	−0.064	0.022	0.063
OOPPaymentsUS	0.004	−0.003	−0.002	−0.003	0.003	−0.002	−0.009	−0.010	−0.005	0.004	−2.72	−11.2	−9.38	−4.55	3.97	−0.003	0.002	0.004	0.002	0.003
PharmaExpendCurr	0.032	−0.023	−0.019	0.031	0.030	0.060	−0.048	0.007	0.123	0.073	123	−26.2	10.7	109	73.2	−0.192	−0.014	−0.079	−0.148	0.078
PharmaExpendUS	0.002	−0.001	−0.003	−0.006	0.003	−0.009	−0.001	−0.003	−0.009	0.004	−7.88	−4.07	−2.61	−6.63	2.39	0.010	0.004	0.005	0.007	0.002
Physicians	0.817	0.105	−0.232	−0.394	0.537	0.060	−0.280	−0.337	−0.621	0.279	−248	−141	−154	−495	164	0.084	0.177	0.186	0.312	0.094
Nurses	0.078	0.248	0.355	−0.230	0.255	0.091	−0.086	−0.002	0.047	0.076	115	−157	16.1	48.3	116	0.226	0.047	0.056	0.152	0.085
MedicalGrads	−0.005	−0.034	−0.023	0.027	0.027	−0.058	−0.076	−0.054	−0.022	0.023	−118	−102	−77.8	−81.0	18.9	0.109	0.094	0.078	0.066	0.019
NursingGrads	−0.000	−0.008	−0.019	−0.011	0.008	0.014	0.008	0.006	0.004	0.004	2.77	10.6	1.77	5.56	3.97	−0.005	−0.004	−0.007	−0.006	0.001
HospitalBeds	0.994	0.616	0.658	0.513	0.208	−0.043	0.167	0.092	−0.109	0.125	−45.6	455	148	−164	270	−0.051	0.121	0.055	0.035	0.169
PsychiatricBeds	1.69	0.135	0.664	4.09	1.75	0.043	0.627	1.28	2.52	1.06	93.1	−775	307	1138	786	−0.076	−0.800	−0.982	−1.79	0.706
MRIUnits	−0.082	−0.022	−0.058	−0.064	0.025	0.005	−0.004	0.000	−0.027	0.014	−7.20	−5.81	−1.56	−44.4	19.9	0.053	0.081	0.045	0.066	0.016
CTScanners	−0.013	0.004	0.016	−0.002	0.012	−0.069	0.003	0.000	−0.017	0.034	−31.6	−1.62	−1.80	−2.35	14.8	0.012	−0.028	−0.010	0.016	0.020
DoctorConsults	−0.168	0.437	0.521	0.586	0.347	0.101	−0.011	−0.014	−0.007	0.056	−58.2	−107	−52.9	−60.6	25.0	0.051	0.119	0.138	0.052	0.045
InpatientDischarges	−0.000	−0.000	−0.000	−0.000	0.000	0.000	−0.000	−0.000	0.000	0.000	0.154	−0.015	0.035	0.188	0.096	−0.000	−0.000	−0.000	−0.000	0.000
AntibioticsDosage	−0.003	0.041	0.066	0.029	0.029	−0.028	0.028	0.002	−0.000	0.023	−8.00	9.39	6.51	0.726	7.67	0.022	0.032	0.023	0.040	0.008
AlcoholConsumption	−0.018	−0.180	−0.117	−0.035	0.075	−0.018	−0.201	−0.191	−0.079	0.089	64.7	−28.7	123	193	93.7	−0.154	−0.284	−0.281	−0.304	0.069
Austria	0.872	5.03	6.00	4.35	2.23	−2.58	1.07	0.961	0.614	1.74	−672	−64.0	383	701	595	1.96	−1.63	−1.09	0.248	1.60
Belgium	−1.97	1.64	1.29	−3.37	2.46	−2.13	−0.700	−1.03	−2.14	0.745	−302	1800	743	711	858	−0.083	−1.93	−1.21	−0.251	0.866
Canada	0.032	1.28	1.82	−2.63	1.98	0.066	−0.165	−0.075	0.230	0.173	−151	232	474	1668	785	−1.14	−2.52	−1.68	−1.44	0.593
Czech Republic	0.479	2.44	2.68	0.86	1.11	−3.03	−2.12	−2.33	−1.90	0.491	−500	721	185	1248	748	−1.16	−2.09	−1.67	−1.34	0.408
Denmark	0.714	3.12	3.33	−0.028	1.69	−3.36	−0.603	−0.862	−1.24	1.26	−889	300	234	902	747	−1.55	−1.23	−1.45	−1.19	0.172
Finland	4.10	5.66	6.04	6.44	1.02	−4.41	−2.41	−2.57	−2.55	0.951	−1331	226	130	727	886	−0.837	−1.67	−1.01	−1.41	0.379
France	1.38	5.80	5.15	1.29	2.40	−2.61	−1.80	−2.28	−2.74	0.420	−2485	−1577	−1753	−1110	571	2.16	0.676	1.73	2.58	0.816
Germany	1.76	4.38	4.38	2.77	1.29	−2.59	−0.764	−1.61	−1.64	0.745	−1318	−395	−770	270	667	−0.719	−2.35	−1.27	−0.862	0.737
Hungary	0.880	0.231	0.970	−1.41	1.10	−2.34	0.899	0.523	0.088	1.46	2542	4435	3575	3332	779	−2.45	−6.21	−4.61	−2.74	1.76
Iceland	−0.702	−1.53	−0.273	0.620	0.895	−3.16	−2.87	−2.85	−1.75	0.621	−721	−94.1	18.3	1607	991	−1.50	−1.57	−1.14	−2.23	0.455
Ireland	0.345	1.25	1.40	−0.173	0.749	−1.03	0.026	−0.644	−1.66	0.707	−633	923	−150	442	679	−2.25	−2.28	−1.33	−0.935	0.672
Japan	NA	18.0	16.9	9.14	4.84	NA	−6.43	−6.77	−4.28	1.35	NA	−2847	−2286	2057	2684	NA	−0.044	1.20	−2.21	1.73
South Korea	6.63	1.74	2.59	−0.260	2.89	−2.83	−3.94	−2.84	−1.25	1.11	1063	−2156	−449	1624	1695	1.33	−2.81	−1.05	−1.94	1.78
Latvia	1.22	0.839	2.16	−1.18	1.41	−1.49	−0.775	−1.29	−0.988	0.317	4786	3584	4415	4404	507	−3.90	−5.69	−4.31	−3.50	0.954
Lithuania	−0.358	−0.133	0.995	0.150	0.592	−2.03	0.509	0.176	0.143	1.16	5192	5953	4948	5152	441	−4.32	−6.53	−4.97	−3.93	1.15
Netherlands	−2.68	1.92	1.33	−7.42	4.32	−4.40	−1.93	−3.01	−4.26	1.16	−2252	−1512	−1251	−150	871	0.350	−1.09	−0.262	0.657	0.772
Poland	−0.750	−0.642	−0.064	−2.90	1.25	−1.73	−1.24	−1.08	−0.497	0.507	1161	961	1642	2436	656	−0.795	−3.78	−2.47	−2.07	1.23
Slovakia	0.218	−0.499	0.236	−2.78	1.42	−0.330	0.632	0.701	1.18	0.632	1929	2665	2813	3212	536	−2.62	−5.43	−4.22	−3.33	1.21
Slovenia	0.442	2.89	3.59	0.371	1.66	−4.92	−2.12	−2.87	−3.62	1.19	−2125	618	142	−118	1209	1.09	−1.23	−0.067	1.24	1.15
Spain	1.07	1.63	2.63	−1.84	1.92	−2.72	−1.26	−0.667	0.147	1.21	207	124	235	1825	820	2.51	0.106	0.644	0.952	1.03

**Table 2:** Results of statistical tests for spatial autocorrelation and order-1 temporal autocorrelation for each response variable.

	$Y_{\mathrm{AverageStay}}$		$Y_{\mathrm{InfantMortality}}$		$Y_{\mathrm{PYLL}}$		$Y_{\mathrm{Life}\;\mathrm{Expectancy}}$	
Test	Statistic	p-Value	Statistic	p-Value	Statistic	p-Value	Statistic	p-Value
Moran’s I	0.94532	0.00	0.68222	0.00	0.89199	0.00	0.90407	0.00
Durbin–Watson	0.47302	1.36 × 10${}^{-34}$	0.96728	2.00 × 10${}^{-18}$	0.70701	1.07 × 10${}^{-24}$	0.69972	1.46 × 10${}^{-26}$
Breusch–Godfrey (AR-1)	119.178	9.57 × 10${}^{-28}$	56.4685	5.71 × 10${}^{-14}$	78.9298	6.44 × 10${}^{-19}$	86.6205	1.31 × 10${}^{-20}$

**Table 3:** Summary of local coefficients estimated through GTWR by dependent variable. (**a**) Average length of stay in inpatient care. (**b**) Infant mortality rate. (**c**) Potential years of life lost. (**d**) Life expectancy of the total population at birth.

**(a)**						
Variable	Min.	1st Qu.	Median	3rd Qu.	Max.	Global (OLS)
Intercept	−2.504 × 10${}^{1}$	−2.252 × 10${}^{-1}$	6.174 × 10${}^{0}$	1.028 × 10${}^{1}$	2.793 × 10${}^{1}$	1.031 × 10${}^{1}$
AlcoholConsumption	−7.897 × 10${}^{-1}$	−8.165 × 10${}^{-2}$	2.376 × 10${}^{-1}$	9.009 × 10${}^{-1}$	3.236 × 10${}^{0}$	−2.646 × 10${}^{-2}$
GovtCompInsUS	−1.006 × 10${}^{-2}$	−1.293 × 10${}^{-3}$	−7.773 × 10${}^{-4}$	2.627 × 10${}^{-4}$	4.900 × 10${}^{-3}$	−5.252 × 10${}^{-4}$
**(b)**						
Variable	Min.	1st Qu.	Median	3rd Qu.	Max.	Global (OLS)
Intercept	2.436 × 10${}^{-1}$	4.517 × 10${}^{0}$	6.294 × 10${}^{0}$	8.082 × 10${}^{0}$	1.866 × 10${}^{1}$	4.921 × 10${}^{0}$
CTScanners	−1.777 × 10${}^{0}$	−2.026 × 10${}^{-1}$	−1.085 × 10${}^{-1}$	−4.323 × 10${}^{-2}$	9.870 × 10${}^{-2}$	−4.485 × 10${}^{-2}$
**(c)**						
Variable	Min.	1st Qu.	Median	3rd Qu.	Max.	Global (OLS)
Intercept	−5.348 × 10${}^{3}$	1.157 × 10${}^{3}$	6.017 × 10${}^{3}$	8.489 × 10${}^{3}$	1.766 × 10${}^{4}$	4.318 × 10${}^{3}$
CTScanners	−2.213 × 10${}^{2}$	−1.585 × 10${}^{2}$	−7.097 × 10${}^{1}$	−1.434 × 10${}^{1}$	5.188 × 10${}^{2}$	−7.525 × 10${}^{1}$
MedicalGrads	−3.978 × 10${}^{2}$	−2.120 × 10${}^{2}$	−7.476 × 10${}^{1}$	−1.031 × 10${}^{1}$	3.930 × 10${}^{2}$	2.809 × 10${}^{1}$
PsychiatricBeds	−7.273 × 10${}^{3}$	−5.597 × 10${}^{3}$	2.348 × 10${}^{3}$	5.710 × 10${}^{3}$	1.896 × 10${}^{4}$	3.267 × 10${}^{3}$
**(d)**						
Variable	Min.	1st Qu.	Median	3rd Qu.	Max.	Global (OLS)
Intercept	6.611 × 10${}^{1}$	7.545 × 10${}^{1}$	7.863 × 10${}^{1}$	8.495 × 10${}^{1}$	9.100 × 10${}^{1}$	8.139 × 10${}^{1}$
MedicalGrads	−3.427 × 10${}^{-1}$	−7.304 × 10${}^{-2}$	1.442 × 10${}^{-1}$	2.140 × 10${}^{-1}$	5.152 × 10${}^{-1}$	−1.330 × 10${}^{-1}$
MRIUnits	−6.570 × 10${}^{-2}$	7.376 × 10${}^{-2}$	1.738 × 10${}^{-1}$	2.583 × 10${}^{-1}$	9.302 × 10${}^{-1}$	2.716 × 10${}^{-1}$
PsychiatricBeds	−2.083 × 10${}^{1}$	−6.992 × 10${}^{0}$	−3.557 × 10${}^{0}$	3.913 × 10${}^{-1}$	8.745 × 10${}^{0}$	−4.960 × 10${}^{0}$

**Table 4:** Comparison of goodness-of-fit across models (the model denoted OLS excludes country dummy variables).

	$Y_{\mathrm{AverageStay}}$			$Y_{\mathrm{InfantMortality}}$			$Y_{\mathrm{PYLL}}$			$Y_{\mathrm{LifeExpectancy}}$		
Model Type	${\mathrm{AIC}}_{c}$	Adj. $R^{2}$	w	${\mathrm{AIC}}_{c}$	Adj. $R^{2}$	w	${\mathrm{AIC}}_{c}$	Adj. $R^{2}$	w	${\mathrm{AIC}}_{c}$	Adj. $R^{2}$	w
OLS	598.0	0.84889	0.000	428.9	0.78320	0.000	2866	0.94639	0.000	467.4	0.95181	0.000
OLS (country)	144.8	0.98696	0.000	243.2	0.92731	0.426	2660	0.98569	0.000	211.2	0.98866	0.000
GTWR	119.4	0.99142	1.000	242.6	0.94010	0.574	2538	0.99485	1.000	144.4	0.99406	1.000

## Data Availability

Publicly available datasets were analyzed in this study. These data can be found here: https://www.oecd.org/els/health-systems/health-data.htm (accessed on 1 March 2021).

## Abbreviations

The following abbreviations are used in this manuscript:

OECD	Organisation for Economic Co-operation and Development
GWR	Geographically weighted regression
TWR	Temporally weighted regression
GTWR	Geographically and temporally weighted regression
CAU	Census area units
MCAR	Missing completely at random
MAR	Missing at random
OLS	Ordinary least squares
RMSE	Root mean squared error
KNN	K-nearest neighbors
MICE	Multiple imputation with chained equations
CC	Complete case analysis
PYLL	Potential years of life lost

## Appendix A. Variables

**Table A1:** List and description of independent variables.

Variable Name	Description
Year	The year in which the data were recorded.
Country	The country from which the data were recorded.
CurrExpendGDP	Current expenditure on health, % of gross domestic product.
CurrExpendUS	Current expenditure on health, per capita, USD purchasing power parities.
GovtCompInsCurr	Government and compulsory health insurance schemes, % of current expenditure on health.
GovtCompInsUS	Government and compulsory health insurance schemes, per capita expenditure, USD purchasing power parities.
OOPPaymentsCurr	Out-of-pocket expenditure, % of current expenditure on health.
OOPPaymentsUS	Out-of-pocket expenditure, per capita, USD purchasing power parity.
PharmaExpendCurr	Current expenditure on pharmaceuticals (prescribed and over-the-counter medicines) and other medical non-durables, % of current expenditure on health.
PharmaExpendUS	Current expenditure on pharmaceuticals (prescribed and over-the-counter medicines) and other medical non-durables, per capita, USD purchasing power parities.
Physicians	Physicians, density per 1000 population.
Nurses	Nurses, density per 1000 population.
MedicalGrads	Medical graduates, per 100,000 population.
NursingGrads	Nursing graduates, per 100,000 population.
HospitalBeds	Total hospital beds, per 1000 population.
PsychiatricBeds	Psychiatric care beds, per 1000 population.
MRIUnits	Magnetic Resonance Imaging units, total, per million population.
CTScanners	Computed Tomography scanners, total, per million population.
DoctorConsults	Doctors consultations, number per capita.
InpatientDischarges	Inpatient care discharges (all hospitals), per 100,000 population.
AntibioticsDosage	Pharmaceutical consumption, antibiotics (J01-Antibacterials for systemic use), defined daily dosage per 1000 inhabitants per day.
AlcoholConsumption	Alcohol consumption, liters per capita (age 15+).

**Table A2:** List and description of dependent variables.

Variable Name	Description
AverageStay	Inpatient care average length of stay (all hospitals), days. Hospitals and the many units within them will have guidelines to meet specific target length of stay for inpatient care.
InfantMortality	Infant mortality (no minimum threshold of gestation period or birth-weight), deaths per 1000 live births. Largely considers the ability for health care systems to support life immediately after birth.
PYLL	Years of life lost across all causes, per 100,000 population, aged 75 years old. This accounts for the potential years of life lost across illnesses including cancers, respiratory diseases, diseases of the blood, diseases of the nervous system, and diseases of the circulatory system, among many others.
LifeExpectancy	Life expectancy, total population at birth, years. Acts as an assessment of the robustness of public health measures, vaccination programs, and gauges the ability for the system to prevent and remedy common ailments as well as environmental factors which may impact health in a person.
